# The role of Ursodeoxycholic acid in non-alcoholic steatohepatitis: a systematic review

**DOI:** 10.1186/1471-230X-13-140

**Published:** 2013-09-23

**Authors:** Zun Xiang, Yi-peng Chen, Kui-fen Ma, Yue-fang Ye, Lin Zheng, Yi-da Yang, You-ming Li, Xi Jin

**Affiliations:** 1 Department of Gastroenterology, The First Affiliated Hospital, College of Medicine, Zhejiang University, Zhejiang, China; 2 Department of Pharmacy, The First Affiliated Hospital, College of Medicine, Zhejiang University, Zhejiang, China; 3 Department of Gastroenterology, The Affiliated Hospital, College of Medicine, Hangzhou Normal University, Hangzhou, China; 4 Department of Infectious Disease, The First Affiliated Hospital, College of Medicine, Zhejiang University, Zhejiang, China

**Keywords:** Ursodeoxycholic acid, UDCA, Non-alcoholic steatohepatitis, NASH, Clinical trial

## Abstract

**Background:**

Non-alcoholic steatohepatitis (NASH) is a condition that occurs during the progression of non-alcoholic fatty liver disease. Effective therapy for NASH is still lacking. In this study, we investigated the effects of Ursodeoxycholic acid (UDCA) in the treatment of NASH.

**Methods:**

Western and Chinese databases were searched by independent investigators using appropriate MESH headings to identify randomized, controlled Western and Chinese clinical trials, published between January 1990 and October 2012, testing the effects of UDCA in patients with NASH. Patient characteristics and trial endpoints were analyzed, with quality assessment according to widely acknowledged criteria. P < 0.05 was defined as statistically significant in all trials.

**Results:**

Twelve qualified randomized clinical trials, including six from China and involving 1160 subjects, were selected. Seven of these trials assessed the effects of UDCA Monotherapy, with the other five testing combinations of UDCA with vitamin E, polyene phosphatidylcholine, silymarin, glycyrrhizin and tiopronin. The duration of therapy ranged from 3 to 24 months, with two studies using high doses of UDCA (23–35 mg/kg/d). The average quality point was 2.69, and was significantly lower in articles from China than in those from Western countries (2.2 ± 0.4 vs. 3.8 ± 1.1, respectively, p < 0.05). UDCA Monotherapy significantly improved liver function in five studies and improved steatosis and fibrosis in two studies. All five studies assessing UDCA combination therapy showed significant improvements liver function, while two studies also improved steatosis and inflammation. One study of high-dose UDCA showed significant improvements in ALT, γGT and liver fibrosis, whereas the other study showed no significant change in ALT and liver pathology.

**Conclusions:**

UDCA therapy is effective in NASH, especially when combined with other drugs. However, the low quality of these studies and the heterogeneity of their results precluded further meta-analysis. Additional carefully designed clinical trials are needed, especially in China.

## Background

Non-alcoholic fatty liver disease (NAFLD), a common pathologic condition characterized by lipid deposition in hepatocytes, can range from simple steatosis to non-alcoholic steatohepatitis (NASH) to fibrosis [1]. NASH occurs in about one quarter of patients with NAFLD [2], indicating disease progression and being a major cause of cryptogenic cirrhosis. A retrospective study showed that 41% of patients with NASH progressed to liver fibrosis and 5.4% to end-stage liver diseases [3]. The prevalence of NASH has increased with increasing obesity and type 2 diabetes, and NASH is currently estimated to affect approximate 1% of the populations of Europe and North America [4]. Despite the “two hit hypothesis” for NAFLD [5], the mechanism by which it progresses to NASH is still vague, but may include oxidative stress, free fatty acid induced lipotoxicity, mitochondrial dysfunction, endoplasmic reticulum stress, dysregulated cytokines and gut bacteria overgrowth [6].

Because of its as yet undetermined pathogenesis, NASH therapy remains empirical and is limited to treating associated conditions, including diabetes, obesity and hyperlipidemia. The current standard of care in the treatment of NASH involves weight loss and increased physical activity, which, while useful in treating simple steatosis, is difficult for patients to achieve [7]. In Western countries, bariatric surgery offers durable weight loss but morbidity rates are high, preventing its further application. Drug regimens are therefore being intensively investigated, with those tested in the treatment of NASH including insulin sensitizers such as thiazolidinediones (TZDs) and metformin, clofibrate, betaine, glucuronate and vitamin E. However, their effects of drugs are confusing and their efficacy unsatisfactory. For example, metformin was not superior to placebo after 6 months in adults [8] and after 24 months in children [9]. Furthermore, a meta-analysis showed that TZDs are associated with weight gain [10], and the cardiovascular safety of glitazones has been questioned [11]. Therefore, finding agents effective in the treatment of NASH is of clinical importance.

Ursodeoxycholic acid (UDCA), a secondary bile acid produced by intestinal bacteria as metabolic by product, has been shown effective in the non-surgical treatment of cholesterol gallstones and primary biliary cirrhosis (PBC) [12]. The clinical properties of UDCA include anti-apoptotic effects, lowering serum TNF-α concentrations, decreasing endoplasmic reticulum stress and improving hepatic insulin sensitivity, suggesting that UDCA may be effective in the treatment of NASH [13]. Clinical trials of UDCA Monotherapy have yielded contradictory results, with higher doses showing marginally positive effects [14]. Trials in China of UDCA therapy in patients with NASH have not been available to Western researchers because of language limitations, and there has been no complete overview of these data. We therefore sought to systematically review the effects of Western and Chinese trials of UDCA in patients with NASH.

## Methods

This systematic review was conducted according to the format of PRIMSA [15] with certain modifications and with the permission of local ethics committee.

### Selection of studies

We searched a combinatory database of MEDLINE, EMBASE, the Cochrane Central Register of Controlled Trials, the Chinese Biomedicine Web Base and Chinese scientific journals’ databases for articles published from January 1990 to October 2012. The search strategy used free-text words and MeSH terms to increase sensitivity, including “UDCA”, “Ursodeoxycholic acid”, “non-alcoholic steatohepatitis” and “NASH”. In addition, citations in retrieved articles were screened and no language restrictions were imposed. Available abstracts from the Digestive Diseases Week and European United Gastroenterology Week conferences were also screened and full texts were requested if necessary. To increase the search scale, experts in the field were also consulted for additional published and unpublished studies. The inclusion criteria for the studies were: 1) original randomized controlled trials published in either Chinese or English, irrespective of blinding; 2) trials reporting, at minimum, changes in liver function or histology; and 3) treatment with a standard or high dose of UDCA, either as Monotherapy or with other drugs.

### Data extraction

Characteristics abstracted from the articles included: the last name of the first author, the year of publication, the location of the study, the number of subject in each group, patient age, drug dose, duration of therapy, study design (randomization method, blinding and number of withdrawals), and concrete data or the effective rate of liver function or histology normalization in patients treated with UDCA. Data from all articles were retrieved by Yue-fang Ye and Shao-hua Chen independently, with the methods and results sections of these studies cut out and coded so that the assessors were blinded to such information. Endpoint outcomes included: liver function improvement, as assessed by alanine aminotransferase (ALT), aspartate aminotransferase (AST) or γ-glutamyl transpeptidase (γGT) concentration and reported as effective rate or real number change; and alleviation in liver histology, as shown by improvements in at least one aspect of steatosis, inflammation or fibrosis, as determined by biopsy or abdominal ultrasound.

### Methodology assessment and statistics

Study eligibility and methodological quality were assessed by three investigators (Zun Xiang, Yi-peng Chen and Kui-fen Ma) independently, with any disagreements resolved by consensus. The methodological quality of included studies was assessed using a five-point quality scale [16] with minor adjustments, including the trial’s design, double blinding, withdrawal rate, analysis and presentation. Numerical variables were compared using Student’s t tests and categorical variables using the chi-square test. P < 0.05 was defined as statistically significant in all analyses.

## Results

### Study design and characteristics

**Figure 1:**
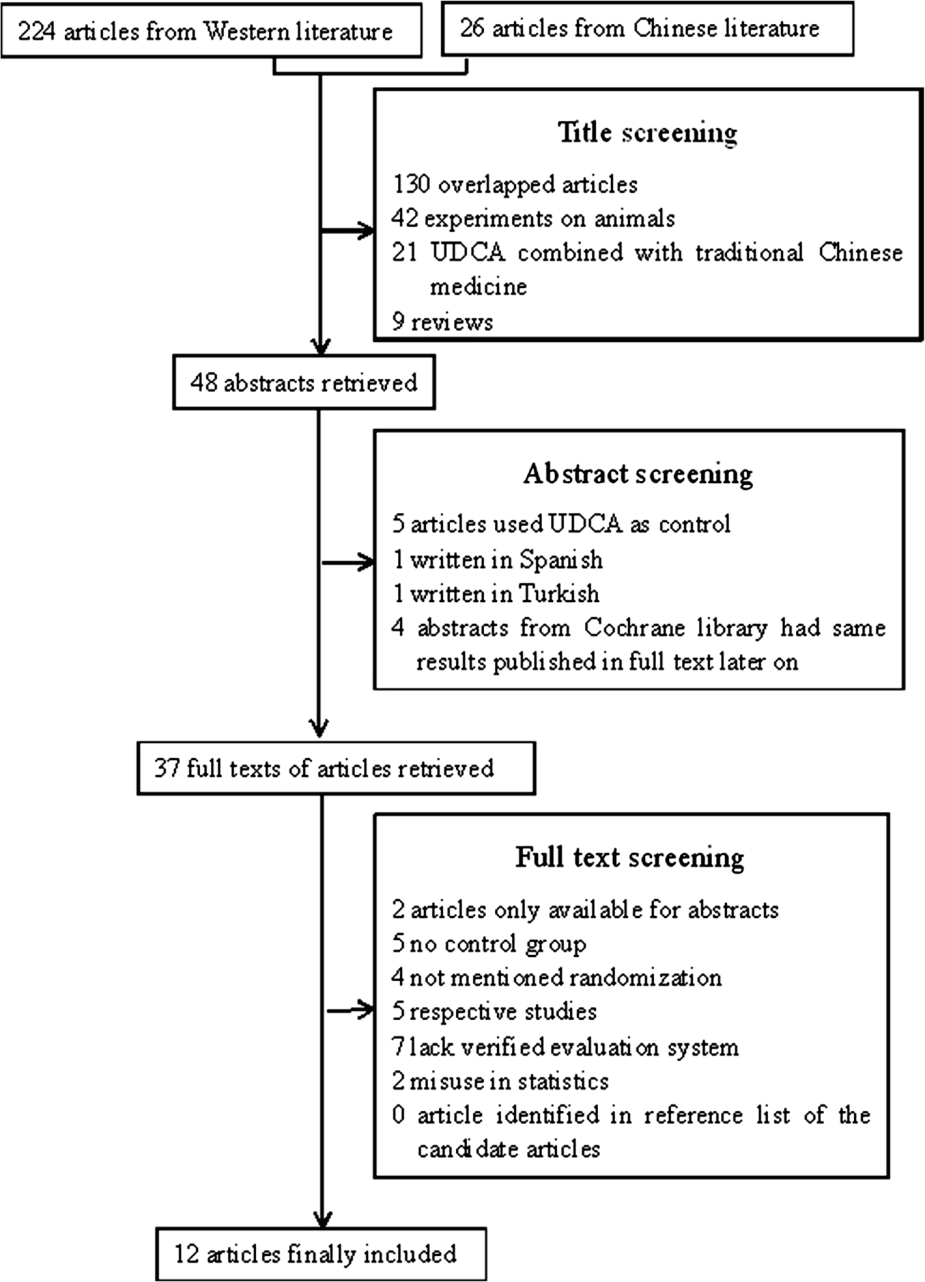
Summary of the article selection process.

A thorough literature search, according to the previously established medical terms, yielded a total of 224 articles from the Western literature and 26 from the Chinese literature. Title screening precluded 176 of these articles (78.6%). The abstracts of the remaining 48 articles were read, and 11 articles were excluded. The full texts of the remaining 37 articles were retrieved. Studies that lacked a control group, verified evaluation system or randomization were excluded (Figure 1). Finally, 12 studies were included, six from Western sources and six from China. These 12 studies could also be categorized into groups by UDCA dosage, or by UDCA Monotherapy or UDCA combined with other drugs. All 12 studies included end-point results presented as concrete data or change in liver function and histology.

The excluded articles included several informative studies. In one excluded study, published in Spanish, three patients were treated with UDCA for 1 year, with one showing ALT and AST normalization [17]. Another excluded study, from the Cochrane Library, showed that UDCA plus pentoxyphylline had significant effects on ALT normalization and hepatic inflammation improvement [18]. A third study showed that UDCA was more effective than gemfibrozil in improving biochemical parameters in patients with NASH patients [19]. However, the full texts of the latter two articles were unavailable. One overlap study used the data from one trial [20] to investigate concentrations of adipokines and apoptosis [21]. Another, which reported that long-term treatment with UDCA and vitamin E significantly improved ALT, AST, and γGT concentrations and NASH score, as well as being well tolerated, had to be excluded because of a lack of a placebo control [22]. Finally, a study comparing the effects of UDCA plus vitamin E with diet and weight management, which found significant ALT normalization in the former group, had to be excluded because of its retrospective design and the absence of histological evaluation after treatment [23].

The 12 included articles [20,24-34] included a total 1160 patients, with average ages ranging from 30.5 to 50.2 years (Table 1). Six studies were from China, one from Turkey and five from Western countries. The trials were performed between 1996 and 2011, with seven trials assessing UDCA Monotherapy and five evaluating UDCA with additional agents, including vitamin E, polyene phosphatidylcholine, silymarin, glycyrrhizin and tiopronin. The duration of treatment ranged from 3 to 24 months. Two studies evaluated high-dose UDCA [28,29] and six used specific drugs instead of placebo as a control [23,25,29-31,33]. One study included three patient groups: UDCA plus vitamin E, UDCA alone and control [20]. Compared with the control group, both treatment groups showed significant ALT improvements, with the UDCA plus vitamin E group also showing alleviation of steatosis. Therefore, the trials were separated into separate categories, including those testing UDCA Monotherapy, UDCA and vitamin E, and UDCA with other drugs.

**Table 1:** Characteristics of the included studies

**Study**	**Year**	**Location**	**Number (age)**^**a**^	**Drug (dosage)**	**Duration**^**b**^
UDCA Monotherapy					
Laurin et al. [24]	1996	USA	25 (46 y) vs. 16 (50 y)	UDCA (13-15 mg/kg/d)/Clofibrate (1 g twice daily)	12 m
Lindor et al. [25]	2004	USA/Canada	80 (45.4 y) vs. 86 (48.5 y)	UDCA (13-15 mg/kg/d)/placebo (tablet)	24 m
Dufour et al. [20]	2006	Switzerland	18 (47 y) vs. 15 (45 y)	UDCA (13-15 mg/kg/d)/placebo (tablet)	24 m
Kiyici et al. [26]	2003	Turkey	17 (43.7 y) vs. 27 (50.2 y)	UDCA (13-15 mg/kg/d)/Atorvastatin (10 mg/d)	6 m
Hong Qian, et al. [32]	2007	China	26 vs. 26	UDCA (15-20 mg/kg/d)/polyene phosphatidylcholine (1368 mg/d)	6 m
Zhu Hong-juan [27]	2010	China	30 (42.5 y) vs. 30 (43.6 y)	UDCA (750 mg/d)/placebo (tablet)	2 m
Ratziu et al. [28]	2011	France	55 (49.8 y) vs. 61 (49.6 y)	UDCA (28-35 mg/kg/d)/placebo (tablet)	12 m
Leuschner et al. [29]	2010	Germany	94 (41.5 y) vs. 91 (45.0 y)	UDCA (23-28 mg/kg/d)/placebo (tablet)	18 m
UDCA combined with other drugs					
Dufour et al. [20]	2006	Switzerland	15 (46 y) vs. 15 (45 y)	UDCA (13-15 mg/kg/d) + Vitamin E (400 IU/d)/placebo (tablet)	24 m
Zhuang Xue-shan [30]	2009	China	40 vs. 42	UDCA (750 mg/d) + polyene phosphatidylcholin (1368 mg/d)/polyene phosphatidylcholine (1368 mg/d)	6 m
Sun Yan [31]	2007	China	76 vs. 61	UDCA (300 mg/d) + Silymarin (231 mg/d)/Silymarin (231 mg/d)	3 m
Lv Hong [33]	2005	China	40 vs. 40	UDCA (450 mg/d) + glycyrrhizin (450 mg/d)/placebo (tablet)	2 m
Liu Zhi-ye [34]	2006	China	96 (42.5 y) vs. 54 (47.6 y)	UDCA (900 mg/d) + Tiopronin (600 mg/d)/ UDCA (900 mg/d)	3 m

^a^number and mean age were displayed as treatment: control group; ^b^duration reported in months; blue indicates a higher dose of UDCA.

### Quality assessment

Because of the stringency of this systematic review, we evaluated the methodological quality of the 12 included trials using a five-point quality scale, as described in the Methods section (Table 2). The average score for all 12 articles was 2.69, with the score being significantly lower for articles from China than for those from Western countries (2.2 ± 0.4 vs. 3.8 ± 1.1, respectively, p < 0.05). Only three articles adequately described the randomization procedure: two from Western countries and one from China (19,27,31). In most articles from Western countries, two to seven patients withdrew from the study, with withdrawals balanced in the treatment and control groups. In the Chinese articles, however, either no patient withdrew or the number was not reported, reducing the quality of these studies. Finally, only four of the 12 studies, all from Western countries, used double blinding. These drawbacks precluded a meta-analysis, resulting instead in a clinical review.

**Table 2:** Methodology quality of the included studies

**Study**	**Randomization**	**Double blinding**	**Withdrawn**	**Total score**
Monotherapy				
Laurin et al. [24]	Inadequate (no description)	No	5 vs. 5	2
Lindor et al. [25]	Inadequate (no description)	Yes	6 vs. 2	4
Dufour et al. [20]	Adequate (randomization list set up by pharmacy before study)	Yes	3 vs. 2	4
Kiyici et al. [26]	Inadequate (no description)	No	NM	2
Hong Qian, et al. [32]	Adequate (1:1 ratio)	NM	0 vs. 0	3
Zhu Hong-Juan [27]	Inadequate (no description)	No	0 vs. 0	2
Ratziu et al. [28]	Adequate (1:1 ratio in blocks of four)	Yes	7 vs. 3	5
Leuschner et al. [29]	Inadequate (no description)	Yes	39 in total	4
UDCA combined with other drugs				
Dufour et al. [20]	Adequate	Yes	3 vs. 2	4
Zhuang Xue-shan [30]	Inadequate (no description)	NM	0 vs. 0	2
Sun Yan [31]	Inadequate (no description)	NM	NM	2
Lv Hong [33]	Inadequate (no description)	NM	NM	2
Liu Zhi-ye [34]	Inadequate (no description)	NM	NM	2

All comparisons were treatment vs. control; *NM* not mentioned.

### Outcome of enrolled studies

The studies tested UDCA Monotherapy or UCDA in combination with other drugs (Table 3). UDCA Monotherapy was found to significantly improve liver function, including ALT, AST or γGT, in five studies [19,23,25-27] and to reduce steatosis and fibrosis in two studies [23,27]. All five studies of UDCA combination therapy found significant improvements in liver function, with two also showing improvements in steatosis and inflammation [19,29]. These data suggested that UDCA combination therapy was superior to UDCA Monotherapy in the treatment of NASH. The results of high-dose UDCA were contradictory, as one study [27] found significant improvements in ALT, γGT and liver fibrosis while the other [28] found no significant changes in ALT and liver pathology, as shown by the Brunt score and NAS score. Finally, improvements in liver function were easier to assess than improvements in liver histology, as eight of 12 studies reported liver function improvement while only four of 12 showed improvements in liver histology.

**Table 3:** End-point results of the included studies

**Study**	**Liver function improvement**	**Histology alleviation**
Monotherapy		
Laurin et al. [24]*	ALT (−30%), ALP (−8%) and γGT (−45%)	steatosis improved
Lindor et al. [25]	ALT in −31% vs. −29%	steatosis in −18% vs. −14%; inflammation in −0 vs. −0.1, fibrosis in 0 vs. 0
Dufour et al. [20]	ALT in −36% vs. −2%	steatosis in −13% vs. −14%, inflammation in −0.8 vs. −0.02, fibrosis in +0.3 vs. +0.4
Kiyici et al. [26]	ALT in −26% vs. −40% ALT in 76.0 vs. 55.1, γGT in 47.8vs 32.2	liver density +20% vs. +35% (in UDCA group between after and before therapy)
Hong Qian, et al. [32]	Effective ratio24/26 vs. 23/27	not mentioned
Zhu Hong-juan [27]	liver function and symptom in 25/30 vs. 15/30	not mentioned
Ratziu et al. [28]	ALT in −28% vs. −2% γGT in −51% vs. +19%	fibrosis in −11% vs. +10%^#^
Leuschner et al. [29]	ALT in −41% vs. −35%	Brunt score in −14% vs. −14% NAS activity score in −21% vs. −18%
UDCA combined with other drugs		
Dufour et al. [20]	ALT in −42% vs. −2% AST in −30% vs. +6%	steatosis in −1.4 vs. −0.5; inflammation in −2.2 vs. −0.8
Zhuang Xue-shan [30]	Effective rate 36/40 vs. 29/42	26/40 vs. 17/42 in steatosis (ultrasound)
Sun Yan [31]	ALT in −79 vs. −72, AST in −31 vs. −8, Effective rate 65/76 vs. 37/61	not mentioned
Lv Hong [33]	ALT in −45.9 vs. −16.1 AST in −40.1 vs. −29.5	no report in histology
Liu Zhi-ye [34]	Effective rate 86/96 vs. 38/54	no report in histology

All comparisons are treatment vs. control. Red indicates statistically significant differences (p < 0.05). *, Data from the UDCA group; the clofibrate group showed no significant changes (not shown) in liver function and histology. ^#^, fibrosis was assessed by FibroTest, not by pathology.

Several studies also found that UDCA alleviated metabolic markers in patients with NASH. For example, patients treated with high-dose UDCA showed significant reductions in serum glucose, glycosylated hemoglobin and insulin concentrations [28], as well a greater ability to reduce triglyceride concentrations than clofibrate [24]. The combinations of UDCA with glycyrrhizin [33] and tiopronin [34] showed additive triglyceride lowering effects when compared with either drug alone.

## Discussion

NAFLD has become the most common chronic liver disease in Western populations, being strongly associated with visceral obesity, insulin resistance, hypertension and hyperlipidemia. NASH, part of the spectrum of NAFLD, was first described in the 1970s in obese females who denied consuming alcohol [35], but generated little interest until the end of 1980s. Because of the limitations of liver biopsy, the true prevalence of NASH is still unclear, although it is currently thought to affect 2–7% of the Western population [36]. NASH has been found to progress to cirrhosis in 10–15% of patients, most frequently after the fifth decade of life [3,37]. Despite its clinical importance, there is still a lack of consensus on NASH treatment. However, the list of potential drugs continues to expand.

UDCA is widely used in the treatment of patients with PBC and primary sclerosing cholangitis (PSC) and has an excellent safety profile. The effect of UDCA in patients with NASH remains unclear because of differences among studies in randomization protocol, inclusion and exclusion criteria, blinding, duration of treatment and combinations with other drugs. Therefore, there is insufficient evidence supporting or refuting UDCA treatment of patients with NASH. We therefore reviewed studies in the Western and Chinese literature, finding 12 randomized, controlled studies investigating the effects of UDCA in patients with NASH. Although three of these articles found that UDCA was ineffective [24,28,31], the other nine found that UDCA had positive effects in patients with NASH, whether as Monotherapy or combined with other drugs. Indeed, our results suggest that UDCA is more effective when combined with other drugs than as Monotherapy.

Although we reviewed 250 articles, we included only 12 in our analysis, a low inclusion rate. This may reflect the relatively wide range of MESH words, our strict selection criteria and the relative dearth of clinical trials in humans. Moreover, the methodological quality of the enrolled studies was variable, being lower in Chinese than in Western studies. This precluded a further meta-analysis, but suggested that Chinese trials require a more stringent study design.

The therapeutic effect of UDCA on NASH is biologically plausible. In an animal model, UDCA was found to improve hepatic steatosis and inflammation [38,39], partly by suppressing the miR-34a/SIRT1/p53 pathway [40]. Although its mechanism of action is still unclear, UDCA can protect hepatocytes by inhibiting the absorption of toxic hydrophobic bile salts from the small intestine, competing with toxic bile acids to bind to cell and organelle membranes and maintain cell membrane stability [41]. In addition, UDCA can reduce oxidative damage by inhibiting hydrophobic bile salt-induced Kupffer cell activation and increasing hepatic glutathione level s [42]. Finally, UDCA has immunomodulatory and anti-apoptotic properties, as shown by its interaction with the glucocorticoid nuclear receptor at the hepatocyte level [43], its repression of IFN-gamma induced MHC class II gene expression [44] and its maintenance of mitochondrial membrane stability [45].

This systematic review and the studies it included had several limitations. First, the possibility of bias and confounders cannot be excluded. Although most studies included stringent criteria for inclusion of patients with NASH and controlled for potential confounders such as age, sex, smoking and alcohol intake, many of these studies could not distinguish between simple steatosis and NASH, especially those studies lacking liver biopsy. Second, UDCA administration and dosage varied widely among studies, as did end point assessments, which were evaluated as actual numbers or as changes in liver function and histology. Third, although six of the 12 studies were performed in China, some were not published in SCI journals, making their credibility, reliability and availability somewhat doubtful. Additional double-blinded randomized clinical trials of UDCA treatment of NASH are urgently needed. Moreover, although we evaluated their methodological quality, some studies had low scores because of the absence of clear descriptions of the method of randomization and the numbers of withdrawals. Fourth, several studies showed ALT decreases in both the treatment and control groups, with the differences not being statistically significant [25,26,29]. Finally, several included papers used liver ultrasound rather than biopsy for assessment, which may impair the strength of these studies.

## Conclusions

In conclusion, the findings from this systematic review indicated that UDCA was useful in NASH therapy, especially when combined with other drugs. However, as the selected studies differed in drug dosage and administration, assessment methods and other aspects, a meta-analysis could not be performed. More stringent studies, especially double-blinded randomized clinical trials, are needed in different countries around the world.

## Competing interests

The authors declare that they have no competing interests.

## Authors’ contributions

XJ formulated the study concept and designed the study. YY and SC retrieved data from all articles independently. ZX, LZ and YY analyzed the research quality and interpreted data. ZX, YC and KM wrote the manuscript. YL critically revised the manuscript for important intellectual content and statistical analysis. All authors read and approved the final manuscript.

## Pre-publication history

The pre-publication history for this paper can be accessed here:

http://www.biomedcentral.com/1471-230X/13/140/prepub
